# Investigating the molecular mechanisms of resveratrol in treating diabetic foot ulcers: a comprehensive analysis of network pharmacology and experiment validation

**DOI:** 10.3389/fmolb.2025.1708426

**Published:** 2025-11-14

**Authors:** Chao Sima, Zhe Wang, Sisi Wang, Haiyang Wang, Zhonghua Sun, Haoguo Wang, Daning Liang, Jianchi Li, Zhenning Zhang

**Affiliations:** 1 Department of Surgery, Shenzhen Guangming District People’s Hospital, Shenzhen, Guangdong, China; 2 Department of Burns, The First People’s Hospital of Zhengzhou, Zhengzhou, Henan, China; 3 Medical School, Nanjing University, Nanjing, Jiangsu, China; 4 Center for Translational Medicine, Guangxi Medical University, Nanning, Guangxi, China

**Keywords:** Diabetic foot ulcers, resveratrol, ScRNA-seq, bioinformatics, molecular docking, machine learning

## Abstract

**Background:**

Diabetic foot ulcers (DFU) are one of the most common and severe complications of diabetes, closely associated with high amputation rates and mortality, and the clinical treatment research is still limited. Previous studies have demonstrated that resveratrol exerts positive effects in wound healing. Therefore, it is necessary to investigate its molecular mechanisms in treating DFU to improve clinical management of this condition.

**Methods:**

This study obtained DFU-related omics data from the GEO database and predicted targets for Resveratrol from TCMSP, PharmMapper, and Swiss Target Prediction. Differential analysis, weighted gene co-expression network analysis (WGCNA), and machine learning were used to jointly identify hub Resveratrol/DFU genes (RDGs). SsGSEA analysis was employed to investigate the relationship between RDGs and the DFU immune microenvironment. Single-cell RNA-seq was employed to investigate cellular heterogeneity of RDGs expression. Molecular docking studies examined interactions between RDGs and resveratrol. Finally, immunohistochemistry validated RDGs expression.

**Results:**

First, bioinformatics analyses and machine learning algorithms identified Cytidine deaminase (CDA) and Ornithine Decarboxylase 1 (ODC1) as RDGs. Second, ROC curves demonstrated RDGs’ strong diagnostic performance for DFU. The ssGSEA algorithm revealed that RDGs partially mediate the immune microenvironment of DFU. Subsequently, scRNA-seq results demonstrated cellular heterogeneity of RDGs expression, which mediates alterations in the pathological microenvironment of DFU and consequently influences its progression. Subsequently, molecular docking revealed strong binding affinity between resveratrol and RDGs, suggesting resveratrol may exert therapeutic effects on DFU by regulating RDG activity through binding. Finally, immunohistochemistry further validated RDG expression, providing strong evidence for RDGs as novel therapeutic targets for DFU.

**Conclusion:**

Overall, this study identified RDGs as a key therapeutic target for resveratrol acting on DFU through a series of bioinformatics analyses and machine learning algorithms. Which not only fills the gap in the molecular mechanism of resveratrol treatment for DFU but also provides a novel therapeutic target for DFU.

## 1 Introduction

Diabetic foot ulcers (DFU) represent the most disabling chronic complication of diabetes, characterized by full-thickness tissue loss in the foot accompanied by persistent inflammation and non-healing wounds, its pathological essence being a cascade amplification effect of neuropathy, vascular disease, and infection induced by a hyperglycemic microenvironment (Jeffc et al., 2024; Armstrong et al., 2023). Currently, DFU treatment poses a formidable challenge to global public health systems. From a health economics perspective, the United States incurs annual direct costs of $273 billion and indirect costs of $90 billion for diabetes care, with foot complications being the primary cost driver, adding 50%–200% in annual additional expenditures (McDermott et al., 2023). Epidemiological data further indicates that approximately one-third of diabetes patients worldwide will develop DFU, with 18.6 million new cases occurring annually (Armstrong et al., 2023). Additionally, approximately 20% of patients with diabetic foot ulcers ultimately require lower limb amputation, with a 5-year mortality rate as high as 50% post-surgery. This severely erodes patients’ quality of life and consumes healthcare resources (Ruder, 2024). Current multidisciplinary management strategies for DFU include surgical debridement, novel wound dressings, interventions targeting lower extremity ischemia, management of foot infections, intravenous antibiotic administration, and alleviation of ulcer weight-bearing pressure, and hyperbaric oxygen therapy (Jeffc et al., 2024; Tan et al., 2024; Everett and Mathioudakis, 2018). Although these approaches promote ulcer healing to some extent, they remain mired in the “high cost-low efficiency-high recurrence” dilemma (Kamaraj et al., 2024). Therefore, identifying natural bioactive molecules that can precisely regulate wound healing while offering both safety and cost-effectiveness has become an urgent task in the clinical treatment of DFU.

Resveratrol is a natural polyphenolic compound widely found in over 70 plant species, including grapes and peanuts (Malaguarnera, 2019; Bi et al., 2023). Resveratrol exhibits multiple significant biological properties, including anti-aging, anti-tumor, anti-inflammatory, anti-oxidative stress, and immunomodulatory effects (Ding et al., 2022; Ren et al., 2021). During DFU treatment, resveratrol can improve the pathological state of diabetic wounds characterized by ‘uncontrolled inflammation and stalled repair’ through multiple mechanisms, including reducing inflammatory infiltration and promoting angiogenesis, thereby accelerating wound healing (Zhu et al., 2022; Zhang et al., 2022; Zhou et al., 2021; Wang A. et al., 2025). DFU wound repair involves multicellular cooperative processes such as fibroblast proliferation, keratinocyte migration, and immune cell polarization. However, how resveratrol modulates the complex networks mentioned above through multi-target, multi-pathway synergistic regulation remains unknown, and its systemic mechanism of action lacks comprehensive elucidation (Ye et al., 2025). Therefore, further investigation into the mechanism of action of resveratrol in the treatment of diabetic foot ulcers is crucial for developing more effective therapeutic strategies.

Network pharmacology, as a core technology in systems biology, constructs interaction networks linking “drug active components-disease targets-signaling pathways.” This enables a holistic analysis of the synergistic therapeutic mechanisms of natural compounds, effectively circumventing the limitations of traditional single-target research (Liang et al., 2025; Hopkins, 2008). This technology demonstrates unique advantages in studying complex diseases driven by multiple factors, such as DFU. By identifying core modular proteins of the disease, it enables precise prediction of synergistic action targets for natural molecules, thereby providing technical support for elucidating the “multi-component-multi-target-multi-pathway” therapeutic model (Chen et al., 2023; Noor et al., 2022; Nogales et al., 2022). Additionally, network pharmacology serves as a core technology in target identification, widely applied in research related to identifying targets for drug treatment of specific diseases and elucidating their molecular mechanisms. For instance, Ji et al. identified key targets for Scutellariae Radix-Coptidis Rhizoma in atherosclerosis through network pharmacology, suggesting its potential multi-component, multi-target, and multi-pathway therapeutic effects against atherosclerosis (Ji et al., 2023). Gu et al. used network pharmacology to identify CX3CR1 as a key target for the Traditional Chinese medicine prescription Sini Decoction in sepsis (Gu et al., 2024). He et al. identified key targets of curcumin in colon cancer through network pharmacology, including CDK2, HSP90AA1, AURKB, etc. (He et al., 2023). Based on this, the present study employs a network pharmacology strategy integrating database mining, bioinformatics analysis, and experimental validation to systematically screen potential core targets and key signaling pathways for resveratrol intervention in DFU. This aims to reveal its multidimensional therapeutic mechanisms and provide theoretical basis for developing novel targeted therapeutic strategies for DFU.

Collectively, the present study identified key therapeutic targets for resveratrol treatment of diabetic foot ulcers through a series of bioinformatics analyses and machine learning algorithms. Specifically, differential expression analysis and WGCNA identified 673 differentially expressed genes (DEGs) associated with diabetic foot ulcers, while multiple databases predicted 391 resveratrol-related targets. The intersection of these two sets revealed 30 overlapping genes. Enrichment analysis revealed these genes primarily participate in immune or inflammation-related pathways, suggesting their potential involvement in the pathogenesis of diabetic foot ulcers. Subsequently, multiple machine learning algorithms identified CDA and ODC1 as RDGs. Then, the single-gene diagnostic ROC curve performance of RDGs demonstrated diagnostic efficacy exceeding 0.9. Single-cell RNA sequencing revealed heterogeneous expression of RDGs in DFU tissues, potentially mediating pathological microenvironment alterations that influence disease progression. Finally, immunohistochemical validation confirmed abnormal expression of RDGs in DFU, providing strong evidence for their potential as novel DFU biomarkers.

## 2 Materials and methods

### 2.1 Acquisition of resveratrol-related targets

This study retrieved the chemical structure and Simplified Molecular Input Line Entry System (SMILES) of resveratrol [SMILES: C1 = CC(=CC = C1/C=C/C2 = CC(=CC(=C2)O)O)O] from the PubChem database (https://pubchem.ncbi.nlm.nih.gov/). Subsequently, the species was specified as “*Homo sapiens*,” and databases such as Traditional Chinese Medicine Systematic Pharmacology (TCMSP, https://old.tcmsp-e.com/tcmsp.php), PharmMapper (https://lilab-ecust.cn/pharmmapper/), and Swiss Target Prediction (http://swisstargetprediction.ch/) to identify potential resveratrol targets (Zhan and Shi, 2025; Yi-Fan and Jian-Rong, 2025; Ru et al., 2014). Next, this study used the Uniprot database to standardize the names of the obtained targets (Hong et al., 2024).

### 2.2 Acquisition of transcriptomic data

The transcriptomic data related to DFU used in this study were downloaded from the GEO database (https://www.ncbi.nlm.nih.gov/geo/). Specifically, we searched the GEO database by typing “Diabetic foot ulcer” and “*Homo sapiens*” as keywords. The datasets were included in the analysis based on the following criteria: (1) the dataset is expected to contain unbiased gene expression data, complete annotation information; (2) the sequencing type should be RNA-Seq; (3) the complete clinical and subgroup information; (4) the data was freely available for download. Based on the above inclusion criteria, we finally selected two datasets, namely GSE134431 and GSE80178. It is worth noting that GSE134431 and GSE80178 have also been employed in previous studies for target identification related to DFU phenotypes, such as glutamine metabolism-related targets, extracellular matrix-related targets, immune-related targets, and exosome-related targets, which further validating the importance and suitability of GSE134431 and GSE80178 for target identification in the present study (Gao et al., 2025; Li et al., 2025; Wu et al., 2024; Shi H. et al., 2024). Meanwhile, we used “ComBat” in the R package “sva” to remove the batch effect from the cohort of GSE134431 and GSE80178 merged (Leek et al., 2012).

### 2.3 Differential analysis of gene expression

In this study, we used the R package “limma” to identify differentially expressed genes (DEGs) between the DFU group and diabetic foot skins (DFS) group (Ritchie et al., 2015). Specifically, we first extracted the expression profiles from the cohort of GSE134431 and GSE80178 merged, and then grouped the samples according to their clinical information. Finally, the DEGs between the two groups were calculated using the R package “limma” with a threshold value of p < 0.05.

### 2.4 WGCNA analysis

We used WGCNA analysis to screen for DFU-related DEGs (Langfelder and Horvath, 2008). Specifically, the data of samples and genes were firstly quality assessed and pre-processed, including detection of missing values, sample clustering, and data matching. Then, the optimal soft threshold was calculated by the network topology analysis function, and the correlation matrix was converted into a weighted adjacency matrix. From the adjacency matrix, a topological overlap matrix is constructed to take topological similarity into account, and a corresponding dissimilarity matrix is built to form clusters. Hierarchical clustering is performed using hclust function utilizing different matrices. Finally, hierarchical clustering was performed based on topological overlap matrices, and the minimum number of genes per module was set to 500 according to the dynamic hybrid cut method, and genes with similar expression patterns were classified into the same module by average association hierarchical clustering. Clinical information was correlated with module characteristic gene expression and gene significance was determined. Then, the correlation of module characteristic genes with DFU was assessed, and modules meeting the study objectives were identified based on the degree of correlation.

### 2.5 Functional enrichment analysis

To explore the biological processes and functions of the resveratrol target-DEGs, we conducted Gene Ontology (GO) and Kyoto Encyclopedia of Genes and Genomes (KEGG) analyses using the R package “clusterProfiler” (Yu et al., 2012), specifying “*Homo sapiens*” as the biological species. All results from this analysis were statistically significant, with a P-value of less than 0.05.

### 2.6 Construction of PPI network

Initially, the biological species was set to “*homo sapiens*” and the 30 resveratrol target-DEGs were imported into the string database, and the interaction score was set to medium confidence (0.400) (https://cn.stringdb.org/) (Szklarczyk et al., 2019). To create a PPI network, the outcomes of the string database were then loaded into the Cytoscape software. The imported genes were then scored using the CytoHubba plugin in Cytoscape (Yu et al., 2023), and the top 20 scoring genes with the highest scores were chosen for further analysis.

### 2.7 Machine learning algorithms

We used multiple machine learning algorithms to identify RDGs, namely, least absolute shrinkage and selection operator (LASSO), random forest (RF) and support vector machine recursive feature elimination (SVM-RFE) (Wang H. et al., 2025). It is worth noting that the combination of these three algorithms plays a crucial role in target identification processes within the biomedical field. They are widely applied in the discovery of novel targets for DFU and various other diseases, such as ulcerative colitis, osteoarthritis, endometriosis, and celiac disease, etc (Tan et al., 2024; Shen et al., 2023; Luan et al., 2023; Shi S. et al., 2024; Guo et al., 2024; Jiang et al., 2024). LASSO is a linear regression method characterized by variable selection and complexity regularization (Vasquez et al., 2016). The present study uses the R package “glmnet” to implement the LASSO analysis and selects the optimal value of λ through a ten-fold cross-validation in order to achieve the best balance between bias and variance. RF utilizes integrated learning to construct multiple decision trees and integrate the prediction results, focusing on evaluating the importance of each variable in the model by rating its importance (Paul et al., 2018). The present study uses the R package “randomForest” to calculate the number of decision trees and the error rate, and when the error rate is stable, the optimal number of decision trees is selected and the candidate genes are ranked in terms of importance. SVM-RFE is a feature selection method based on support vector machines, where all the features are trained and evaluated through multiple iterations using support vector machines in each iteration (Noble, 2006). The present study used the R package “e1071” for SVM-RFE analysis, and selected key features by performing ten-fold cross-validation and weighted summing of the number of gene occurrences and the order of importance. Then, we used venn diagrams to intersect the candidate genes screened by the above three algorithms as hub Resveratrol/DFU genes (RDGs).

### 2.8 Evaluation of diagnostic performance of RDGs

To evaluate the diagnostic performance of RDGs in DFU, we employed receiver operating characteristic (ROC) analysis using the “pROC” library in R. The “roc” function was utilized to generate the curves, while the “ci” function computed the final area under the ROC curve (AUC) values.

### 2.9 Immune cells infiltration analysis

The present study used ssGSEA to assess immune cell infiltration (Hänzelmann et al., 2013). Specifically, we performed ssGSEA analysis in R, employing the “GSVA” and “GSEABase” packages to evaluate the immunological profiles of DFU patients. To compare the abundance of various immune cell infiltrates between DFS group and DFU group, the Wilcoxon test was applied for statistical comparisons. Following this, the “ggplot” package in R was employed to graphically represent the relationship between immune cell infiltration levels and RDGs expression.

### 2.10 scRNA-seq analysis

The DFU-related scRNA-seq used in this study was obtained from the previous work of Theocharidis et al. (2022), and analyzed using the R software package “Seurat” (Satija et al., 2015). Specifically, this study was analyzed using the following process: (1) Filter out low-quality cells using the following thresholds: nFeature_RNA >300, nFeature_RNA <7500, mt_percent <10; (2) “NormalizeData” was used to standardize expression levels; (3) “ElbowPlot” was used to determine the PCA dimension, and then the top 20 principal components were extracted; (4) cell clustering using “FindNeighbors” and “FindClusters”; (5) dimensionality reduction visualization using “RunUMAP”; (6) annotation of cell subpopulations based on known marker genes (Theocharidis et al., 2022; Reynolds et al., 2021; Lu et al., 2023; He et al., 2020).

### 2.11 Molecular docking

Molecular docking is a common method used in drug discovery to accurately predict protein binding sites and small molecule ligand conformations, and to assess the binding affinity between them (Xiao et al., 2024). Specifically, we firstly downloaded the small molecule ligand files from the PubChem database (http://pubchem.ncbi.nlm.nih.gov/). We downloaded the PDB files of RDGs target proteins from the PDB database (https://www.rcsb.org/), removed water molecules, small-molecule ligands using the Pymol software, and hydrogenated, calculated charges, and set rigid molecules using the AutoDocKTools software. Subsequently, AutoDocKTools software was run to hydrogenate the target protein and other processes, and the target protein and ligand small molecules were converted to pdbqt format, respectively. Finally, a command prompt was run to dock the target protein and ligand as well as to calculate the binding energy.

### 2.12 Patient screening and clinical specimen collection

The specimens were obtained from diabetic patients admitted to Shenzhen Guangming District People’s Hospital who met the following criteria. This study enrolled patients with diabetic foot skin (DFS) and diabetic foot ulcers (DFU). The inclusion criteria were as follows: meeting the diabetes diagnostic criteria established by the World Health Organization (WHO), being aged between 18 and 75 years, and voluntarily signing an informed consent form. Patients with the following conditions were excluded: severe uncontrolled systemic infection, allergy to operations related to specimen collection, severe liver function abnormalities, malignant tumors, New York Heart Association (NYHA) cardiac function class Ⅲ-Ⅳ, coagulation disorders, being pregnant or lactating, and having mental illnesses. Specimen collection strictly followed aseptic operation procedures. Residual DFS skin tissues after patients underwent operations (such as debridement) and skin tissue specimens from the edges of DFU ulcers after surgical treatment were collected.

### 2.13 Immunohistochemistry

Immunohistochemical experiments were performed on the collected postoperative skin tissue specimens of DFS and DFU: the specimens were fixed with 4% paraformaldehyde, embedded in paraffin, and sectioned. After dewaxing to water, antigen retrieval was performed with citrate buffer (pH 6.0) by heat. Then, they were incubated with 3% hydrogen peroxide at room temperature for 10 min to block endogenous peroxidase activity; after blocking with 5% bovine serum albumin at 37 °C for 30 min, ODC1 antibody (1:100, Zenbio, China) and CDA antibody (1:100, Zenbio, China) were added dropwise respectively and incubated overnight at 4 °C; after rewarming the next day, they were washed with PBS 3 times (5 min each time), then HRP-labeled secondary antibody (1:500) was added dropwise and incubated at 37 °C for 60 min. After washing again, the color reaction was carried out using a DAB chromogenic kit, followed by hematoxylin counterstaining of cell nuclei. After gradient ethanol dehydration and xylene transparency, the sections were sealed with neutral gum. Observe through an inverted microscope.

### 2.14 Statistical analysis

In this study, the statistical analysis and visualization were performed in R language, and the Wilcoxon rank sum test was used to compare the two groups, with P < 0.05 indicating a statistically significant difference.

## 3 Results

### 3.1 Identification of differentially expressed genes in DFU

**FIGURE 1:**
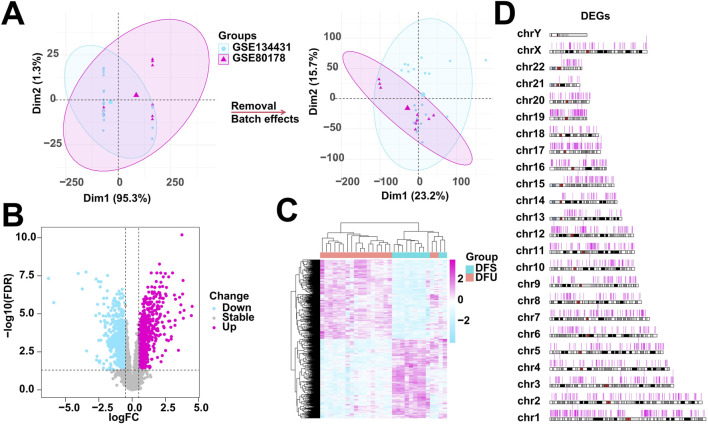
Data processing and identification of DEGs. **(A)** Elimination of batch effects in the training cohort (GSE134431 and GSE80178). **(B)** Volcano diagram showing DEGs between the two subgroups. **(C)** Heatmap showing the overall landscape of DEGs expression between the two subgroups. **(D)** Chromosome map showing the distribution of DEGs on chromosomes.

In this study, GSE134431 and GSE80178 were used as training cohorts. However, there are batch effects between the different datasets included in this study due to different sequencing platforms, etc. Therefore, we need to eliminate the batch effects in the training cohort for subsequent analysis. We used the Combat function in the R package “sva” to remove the batch effect in the training cohort after combining GSE134431 and GSE80178. The result after removing the batch shows that the samples are evenly dispersed and can be used for subsequent analysis (Figure 1A). Immediately after that, we used the RNA-seq of the training cohort as the expression profile, combined with the clinical information of the samples, that is, DFSs and DFU as two different subgroups for differential gene expression analysis. Eventually, we obtained 1727 DEGs, of which 864 were downregulated expressed genes and 863 were upregulated expressed genes (Figure 1B), and the overall expression landscapes of these DEGs were visualized as shown in Figures 1C,D, that is, the expression was obviously different between the two groups and evenly dispersed on each chromosome.

### 3.2 Identification of DFU-related DEGs through WGCNA analysis

**FIGURE 2:**
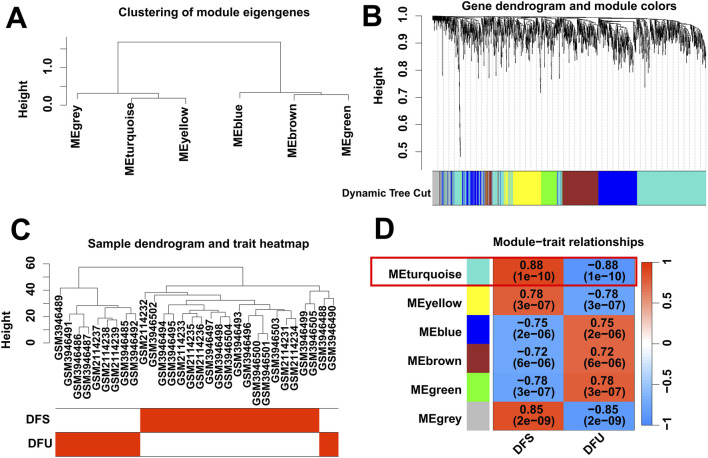
Identification of DFU-related DEGs by WGCNA. **(A,B)** Clustering dendrogram of DEGs. **(C)** Clustering dendrogram of DFU and DFS samples. **(D)** Heatmap of correlation between module DEGs and phenotypes.

Gene expression is associated with a diverse range of signaling pathways and biochemical responses in the body, and abnormalities in these pathways and responses can lead to the development of a variety of diseases in certain conditions. To identify DFU-related DEGs, we performed WGCNA analysis based on RNA-seq from the training cohort. Specifically, we categorized the genes into six independent co-expression modules based on their expression patterns in the training cohort (Figures 2A,B). Subsequently, we combined the clinical information of the samples in the training cohort, and the results showed that the DFU samples were highly distinguishable from the DFS samples (Figure 2C). The correlogram of module-trait relationships showed that the turquoise module, which contains 673 DEGs, had the highest correlation with DFU (Figure 2D).

### 3.3 Identification of resveratrol target-genes dysregulately expressed in DFU

**FIGURE 3:**
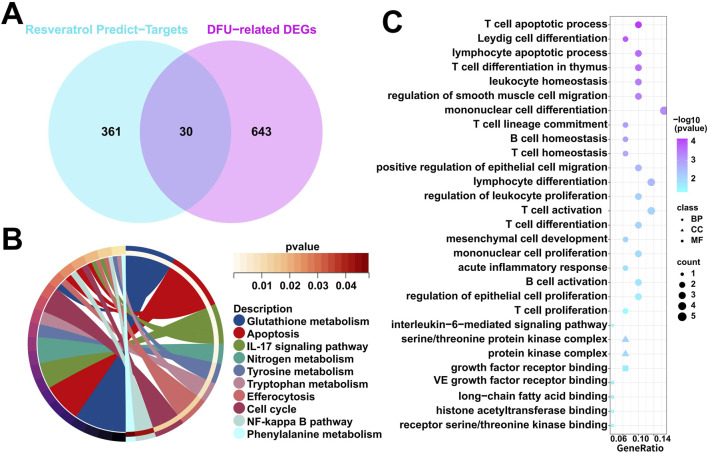
Characterization of resveratrol target-genes dysregulately expressed in DFU. **(A)** Venn diagram showing overlapped genes between the DFU-related DEGs and the resveratrol target-genes. **(B)** KEGG enrichment results in the overlapped genes. **(C)** GO enrichment results in the overlapped genes.

Firstly, we obtained 391 resveratrol predict-targets through the TCMSP, PharmMapper and Swiss Target Prediction databases. Immediately after that, we cross-analyzed the 673 DFU-related DEGs identified in the above results with the 391 resveratrol predict-targets obtained in this study, resulting in 30 overlapped genes (Figure 3A). Subsequently, we performed functional enrichment analysis on these 30 overlapped genes, in which KEGG enrichment analysis showed that they were related to immune/metabolism, such as “IL-17 signaling pathway”, “NF-kappa B signaling pathway”, “Glutathione metabolism”, “Nitrogen metabolism”, “Tyrosine metabolism”, “Tryptophan metabolism”, and “Phenylalanine metabolism” (Figure 3B). The results of GO enrichment analyses were similar to those of KEGG enrichment analyses, showing that they are mainly associated with immune/inflammatory responses, such as “leukocyte homeostasis”, “B cell homeostasis”, “T cell homeostasis”, “T cell activation”, “acute inflammatory response”, “B cell activation”, “T cell proliferation”, “interleukin-6-mediated signaling pathway (Figure 3C). In summary, the pathogenesis of DFU may be related to the metabolism pathway and the immune/inflammatory response.

**FIGURE 4:**
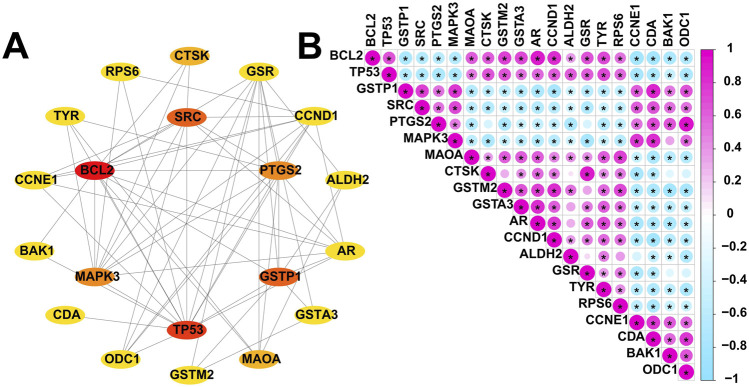
Expression patterns of resveratrol target-DEGs in DFU patients. **(A)** The protein-protein interaction network showing the interactions between the top 20 scoring genes. **(B)** Correlation analysis of the top 20 scoring genes. Red and green colors represent positive and negative correlations, respectively. (*p < 0.05).

Certainly, the identification of key genes is determined by multiple factors. In addition to the analysis of gene expression patterns mentioned above, the interaction between genes cannot be ignored. Specifically, we included these 30 overlapped genes in the PPI analysis. We evaluated these 30 overlapped genes comprehensively in Cytoscape using CytoHubba plugin and selected the top 20 scoring genes with the highest scores (Figure 4A). Given the functional interdependence between genes, we conducted correlation analysis on the top 20 highest-scoring genes. The results revealed significant co-expression patterns among these genes (Figure 4B), indicating that the dysregulated expression of resveratrol targets in DFU forms a coordinated network that likely contributes to DFU pathogenesis and progression.

### 3.4 Identification of RDGs based on multiple machine learning algorithms

**FIGURE 5:**
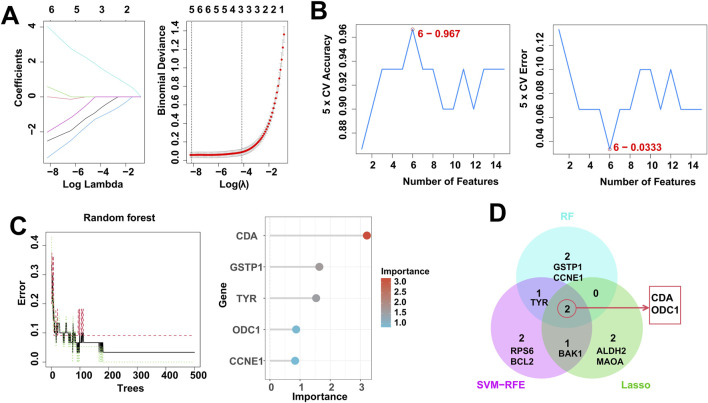
Identification of RDGs by multiple machine learning. **(A)** LASSO regression algorithm to screen candidate genes. **(B)** SVM-RFE algorithm for screening candidate genes. **(C)** RF algorithm for screening candidate genes. **(D)** Venn diagram showing the intersection of candidate genes obtained by the 3 machine learning algorithms.

In order to identify RDGs that play a pivotal role in the pathogenesis of DFU, we applied SVM-RFE, LASSO and RF to analyze and identify the top 20 scoring genes. Specifically, we obtained 5 candidate genes by LASSO algorithm, including ODC1, ALDH2, MAOA, CDA, and BAK1 (Figure 5A), 6 candidate genes by SVM-RFE algorithm, including CDA, TYR, BAK1, RPS6, BCL2, and ODC1 (Figure 5B), and 5 candidate genes by RF algorithm, including CDA, GSTP1, TYR, ODC1, and CCNE1 (Figure 5C). Then, the candidate genes identified by the above three machine learning algorithms were taken to intersect, resulting in two hub Resveratrol/DFU genes (RDGs), namely, CDA and ODC1 (Figure 5D).

### 3.5 Diagnostic performance of RDGs for DFU

**FIGURE 6:**
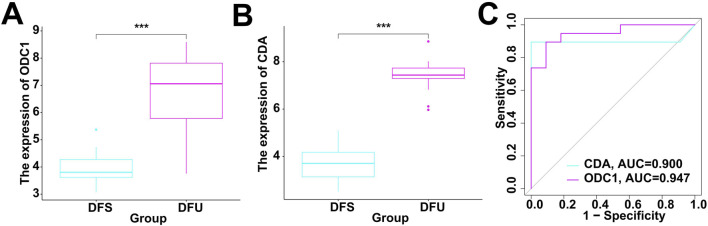
Evaluation of diagnostic performance of RDGs. **(A)** The expression of ODC1 between the DFS and DFU. **(B)** The expression of CDA between the DFS and DFU. **(C)** ROC showed the diagnostic performance of the RDGs.

The above-mentioned studies indicate that RDGs are key targets for resveratrol in DFU. Therefore, it is necessary to investigate whether they have the potential to be used as diagnostic targets for DFU. Specifically, this study integrated the included bulk RNA-seq data to investigate the expression levels of RDGs. The results showed that RDGs were significantly upregulated in DFU (Figures 6A,B), which provides strong evidence for their use as diagnostic targets for DFU. Subsequently, this study used RDGs for single-gene diagnostic analysis of DFU, with DFS and DFU as diagnostic binary variables. ROC results showed that the diagnostic ROC values of RDGs for DFU all exceeded 0.9 (Figure 6C), indicating that RDGs have the potential to become novel diagnostic targets for DFU.

### 3.6 Immune cell infiltration analysis

**FIGURE 7:**
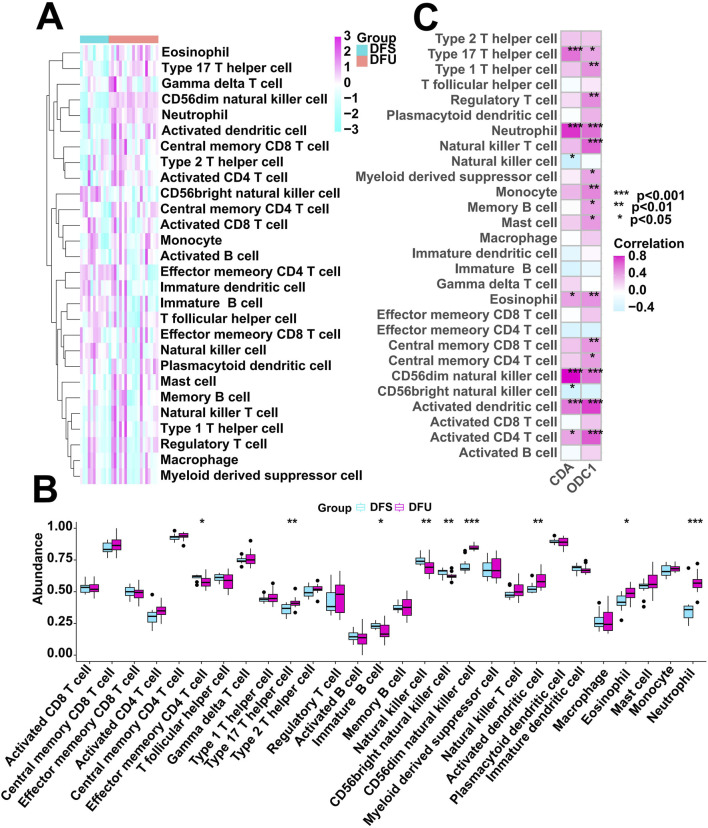
Immune cell infiltration analysis. **(A)** Heatmap showing the overall landscape of immune cell abundance. **(B)** Box diagram showing the differences in immune cell infiltration abundance between the DFS and DFU. **(C)** Correlation analysis between the expression of RDGs and immune cell infiltration abundance. (*p < 0.05, **p < 0.01, ***p < 0.001)

We utilized the ssGSEA algorithm to assess the immunological characteristics of the DFU samples. Figure 7A shows the overall immune cell infiltration between the two groups of DFU samples and DFS samples, and the results indicate that there is a significant difference in immune cell infiltration between these two groups. The abundance of immune cell infiltration was significantly higher in DFU patients compared to DFS samples, and the immune infiltration analysis showed that a total of 5 types of immune cells were more abundant in the DFU group than those in the DFS group, including Type 17 T helper cell, CD56dim natural killer cell, Activated dendritic cell, Eosinophil, and Neutrophil (Figure 7B). This suggests that the immune microenvironment disturbance may also be a contributor to the development of DFU. Certainly, it is necessary to explore the relationship between the dysregulated expression of RDGs and the immune microenvironment. Next, we investigated the relationship between the expression of RDGs and the abundance of immune cells by correlation analysis. The result showed a significant correlation between the expression of RDGs and the abundance of a variety of immune cells (Figure 7C), which suggests that RDGs may have a potential role in regulating the DFU immune microenvironment.

### 3.7 ScRNA-seq profiling analysis of DFU

**FIGURE 8:**
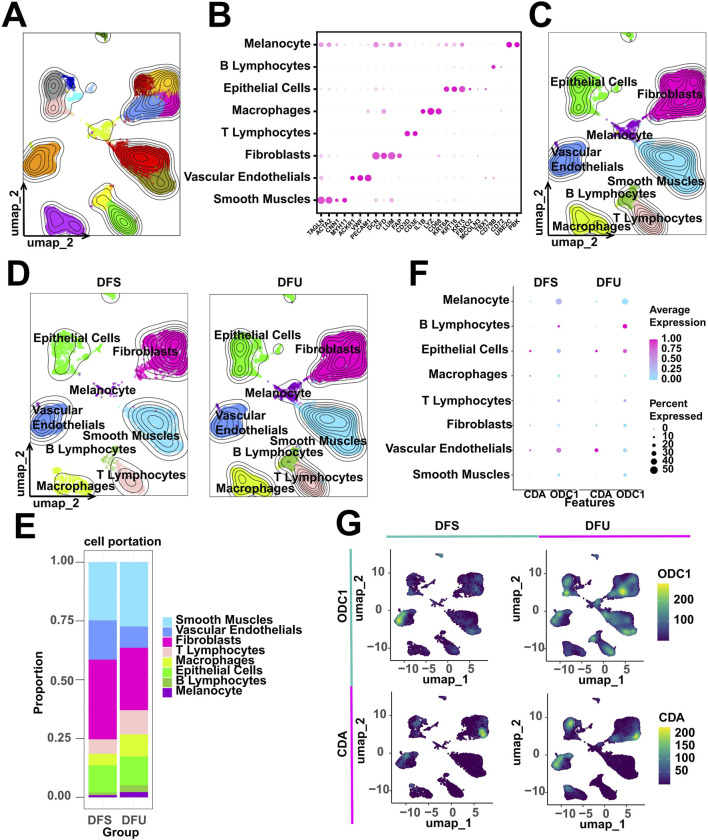
scRNA-seq analysis of DFU patients. **(A)** 17 cell clusters were identified. **(B)** Expression dot plots of known markers to support cell annotation. **(C,D)** 8 celltypes were annotated. **(E)** Counting cell proportions. **(F)** Grouping expression dot plots of RDGs. **(G)** Grouping expression heatmap of RDGs.

Initially, we performed clustering analysis on the scRNA-seq data obtained from DFU patients using the UMAP algorithm, which delineated 17 clusters (Figure 8A). These clusters were subsequently annotated based on known markers (Figure 8B). Our scRNA-seq profiling identified a total of eight major celltypes, with their respective single-cell transcriptomic landscapes illustrated in Figure 8C. To assess disease-associated cellular heterogeneity, we stratified the single-cell data by clinical status (Figure 8D). Cell proportion analysis demonstrated a significant enrichment of smooth muscle cells, T/B lymphocytes, and macrophages in DFU samples (Figure 8E), suggesting that this altered cellular distribution may contribute to the chronic inflammatory and ulcerative microenvironment characteristic of DFU. Given the potential involvement of RDGs in disease pathogenesis, we further investigated their expression patterns at single-cell level. Notably, RDGs exhibited elevated expression levels in DFU patients (Figure 8F), consistent with the dysregulation observed in bulk RNA-seq analyses. Expression heatmap confirmed detectable RDG activity across multiple cell types, with prominent enrichment in vascular endothelial cells, epithelial cells, and fibroblasts (Figure 8G). Collectively, these findings highlight the heterogeneous expression of RDGs at single-cell level, reinforcing their potential as key regulators in DFU progression and therapeutic targets for intervention.

### 3.8 Molecular docking between resveratrol and RDGs

**FIGURE 9:**
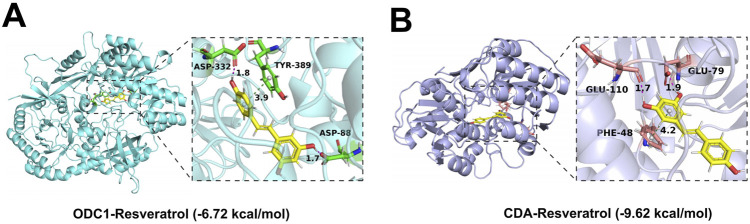
3D views of the interacted interface between RDGs and resveratrol. **(A)** The structure of the complex formed by the docking of resveratrol with ODC1. **(B)** The structure of the complexes formed by the docking of resveratrol with CDA.

This study conducted comprehensive molecular docking simulations to further elucidate the interactions between resveratrol and RDGs. Specifically, RDGs was used as the receptor and resveratrol as the ligand, and AutoDock software was employed to generate docking results for them. The molecular docking results indicated that RDGs exhibit binding capacity with resveratrol (binding energy <0 kcal/mol). Specifically, the results showed that the binding affinities of resveratrol docked to RDGs were −6.72 kcal/mol (Target: ODC1) and −9.62 kcal/mol (Target: CDA), respectively (Figures 9A,B). Altogether, the docking results suggest that resveratrol may be able to interact with RDGs with higher binding energies and thus achieve therapeutic or palliative effects on DFU symptoms.

### 3.9 The expression of RDGs in human DFU samples

**FIGURE 10:**
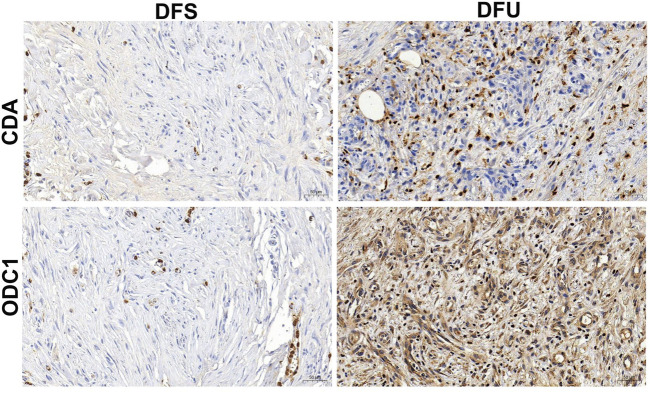
Immunohistochemical staining of RDGs from human’s DFU samples and DFS samples.

The above-mentioned study has demonstrated from multiple dimensions that RDGs are the hub targets of resveratrol acting on DFU. Certainly, the significance of RDGs still needs to be verified through experiments. The analysis results of bulk RNA-seq of DFU showed that RDGs were significantly upregulated in DFU compared with DFS. Therefore, in this study, the expression of RDGs was verified by combining clinical DFU samples and using IHC experiments. Specifically, the results of IHC indicated that RDGs were highly expressed in the clinical tissues of DFU (Figure 10), which was consistent with the above transcriptome analysis results and further provided strong evidence for the hub targets of this study. Taken together, these results suggested the potential role of RDGs in the pathogenesis of DFU.

## 4 Discussion

Diabetic foot ulcers (DFU) represent one of the most serious and prevalent complications of diabetes, defined as chronic, non-healing wounds occurring on the lower limbs of diabetic patients (Armstrong et al., 2023; Jeyam et al., 2020). Epidemiological studies indicate that DFU constitute a significant global public health burden. Currently, 15%–25% of diabetic patients will develop foot ulcers during their lifetime, and nearly 20% of patients ultimately require lower limb amputation due to uncontrolled infection or tissue necrosis (Xia et al., 2025; Zhao et al., 2025). Beyond physical suffering, foot ulcers impose a substantial socioeconomic burden, further diminishing patients’ quality of life (Rathnayake et al., 2020). Clinically, DFU present major challenges in both diagnosis and treatment. At present, the clinical diagnostic workflow for DFU primarily centers on the assessment of lower limb vasculopathy and peripheral neuropathy (Sloan et al., 2021). The diagnosis of lower extremity vasculopathy relies on the fulfillment of the following diagnostic parameters: (1) confirmation of diabetes mellitus diagnosis; (2) presence of clinical signs and symptoms indicative of lower extremity ischemia; (3) auxiliary examination results suggesting lower extremity vasculopathy. The diagnosis of peripheral neuropathy is guided by the presence of the following abnormal findings: (1) disturbed thermal sensation; (2) diminished or absent plantar sensation detected via nylon monofilament testing; (3) abnormal vibratory perception; (4) absent ankle reflexes; (5) slowing down of 2 or more items of nerve conduction velocity (Tan et al., 2024). Conventional clinical assessments lack sufficient sensitivity to subtle changes in the wound microenvironment, hindering early diagnosis. Meanwhile, advanced diagnostic tools such as imaging or biomarker testing remain underutilized in primary care settings (Zhou et al., 2022). Traditional therapies, including surgical debridement, novel wound dressings, interventions targeting lower extremity ischemia, management of foot infections, intravenous antibiotic administration, and alleviation of ulcer weight-bearing pressure, and hyperbaric oxygen therapy—continue to have inherent limitations (Jeffc et al., 2024; Tan et al., 2024; Everett and Mathioudakis, 2018). Even advanced approaches like negative pressure wound therapy remain limited by high costs, technical complexity, and inconsistent efficacy across patient subgroups (Bandyk, 2018; Shu et al., 2018). Given these unmet clinical needs, there is an urgent requirement to develop novel therapeutic agents targeting the multidimensional pathophysiological mechanisms of diabetic foot ulcers.

Resveratrol is a natural polyphenolic compound widely found in plants such as grapes and peanuts. It has garnered significant attention for its diverse biological activities, including anti-aging, anti-tumor, anti-inflammatory, anti-oxidative stress, and immune-modulating effects (Malaguarnera, 2019; Bi et al., 2023; Ding et al., 2022; Ren et al., 2021). In the treatment of diabetic foot ulcers (DFU), studies have demonstrated that resveratrol can improve the pathological state of ‘inflammatory dysregulation - repair arrest’ in diabetic wounds through multiple pathways. These include reducing oxidative stress, alleviating inflammatory infiltration, promoting angiogenesis, and inhibiting ferroptosis, thereby accelerating wound healing (Zhu et al., 2022; Zhang et al., 2022; Zhou et al., 2021; Wang A. et al., 2025). However, DFU wound repair involves a multi-cellular cooperative process encompassing fibroblast proliferation, keratinocyte migration, and immune cell polarization. The precise molecular mechanisms by which resveratrol modulates this complex biological network through multi-target, multi-pathway regulation to exert therapeutic effects remain to be ascertained, necessitating systematic and comprehensive in-depth research.

To elucidate the multidimensional mechanisms of resveratrol in treating DFU, this study integrated a series of bioinformatics techniques and experimental methods. First, DFU-related transcriptome data were obtained from the GEO database, and the R package “limma” identified 1,727 differentially expressed genes (DEGs) in DFU, comprising 864 downregulated genes and 863 upregulated genes. Subsequently, weighted gene co-expression network analysis (WGCNA) was employed to mine DFU-associated DEGs. To ascertain potential target sites for resveratrol in DFU, predicted targets for resveratrol were first obtained from the TCMSP, PharmMapper, and Swiss Target Prediction databases. These were then cross-analyzed with DFU-related DEGs to identify overlapping genes. Subsequently, functional enrichment analysis of these overlapping genes using the R package “clusterProfiler” revealed their primary involvement in immune/metabolic pathways (e.g., IL-17 signaling, NF-κB signaling, glutathione metabolism) and immune/inflammatory responses (e.g., leukocyte homeostasis, T/B cell activation, acute inflammatory response). This provides crucial insights into the potential mechanisms of resveratrol in DFU. To further identify core targets, this study employed multiple machine learning algorithms, ultimately identifying two RDGs: Cytidine deaminase (CDA) and Ornithine Decarboxylase 1 (ODC1).

CDA and ODC1 have been identified as RDGs, a finding supported by previous evidence. These studies indicate that both genes are closely associated with wound healing, inflammation regulation, and metabolic processes—core mechanisms in the pathogenesis of DFU (Durr et al., 2025). As a key enzyme in pyrimidine metabolism, CDA plays a vital role in regulating nucleotide homeostasis and immune cell function (Ligasová et al., 2025; Lv et al., 2025). In chronic wounds like DFU, disrupted nucleotide metabolism interferes with immune cell activation and proliferation, thereby hindering inflammation resolution and tissue repair (Liao et al., 2025). Previous studies have revealed that CDA expression undergoes alterations in inflammatory states, with changes in its activity influencing the balance between pro-inflammatory and anti-inflammatory mediators (Lu et al., 2023). For example, in skin inflammation models, CDA deficiency leads to enhanced inflammatory cell infiltration and delayed wound healing, fully demonstrating its role in regulating the inflammatory microenvironment (Reynolds et al., 2021; Naso et al., 2023). ODC1 is the rate-limiting enzyme in polyamine biosynthesis, and polyamines are crucial for cell proliferation, migration, and tissue regeneration—all key processes in wound healing (Gao et al., 2024; Bhalla and Lee, 2025). Studies indicate that dysregulated ODC1 expression in diabetic wounds leads to reduced polyamine levels, resulting in impaired fibroblast function and decreased keratinocyte migration (He et al., 2020; Li et al., 2024). In diabetic animal models, restoring ODC1 activity or supplementing polyamines accelerates wound healing by promoting cell proliferation and angiogenesis, highlighting ODC1 as a potential therapeutic target for DFU (Theocharidis et al., 2022; Kaur et al., 2025). Collectively, these findings indicate that CDA and ODC1 not only participate in core pathological processes of DFU but may also serve as potential therapeutic targets.

In this study, we further confirmed that CDA and ODC1 hold potential as diagnostic markers and therapeutic targets for DFU, along with their interactive relationship with resveratrol. To elaborate, receiver operating characteristic (ROC) analyses were performed using the R package “pROC”. The findings showed that both CDA and ODC1 displayed superior diagnostic capabilities for DFU, with area under the ROC curve (AUC) values surpassing 0.9. This high AUC indicates their strong ability to differentiate between DFU and DFS samples, thereby offering robust support for their potential as new diagnostic biomarkers for DFU (Guo et al., 2024). Moreover, molecular docking experiments conducted via AutoDock software demonstrated that resveratrol has a strong binding capacity with these two key regulatory genes (RDGs). Specifically, the binding energies of resveratrol with ODC1 and CDA were −6.72 kcal/mol and −9.62 kcal/mol, respectively. Both values being below 0 kcal/mol suggests the presence of stable intermolecular interactions between resveratrol and these two proteins (Shen et al., 2023; Xiao et al., 2024; Aguiar and Camps, 2024). These results imply that resveratrol might exert its therapeutic effects on DFU by binding to CDA and ODC1, thereby modulating their biological activities.

Although this study obtained several valuable findings, it must be acknowledged that certain limitations remain. First, while multiple bioinformatics methods and machine learning algorithms were employed to identify and validate CDA and ODC1 as RDGs, the specific functional mechanisms underlying the interaction between resveratrol and these two genes require further elucidation through in-depth *in vitro* experiments. Second, while immunohistochemical staining confirmed the expression of CDA and ODC1 in clinical diabetic foot ulcer samples, the limited sample size necessitates larger cohort studies to validate their diagnostic value and clinical significance. In summary, subsequent research should integrate *in vitro* and *in vivo* experiments to ascertain the precise mechanisms by which resveratrol regulates CDA and ODC1.

## 5 Conclusion

In summary, this study identified RDGs as key therapeutic targets for resveratrol in treating DFU through a series of bioinformatics analyses and machine learning algorithms, and validated the importance of RDGs for DFU across multiple dimensions. This research not only elucidates the molecular mechanism underlying resveratrol therapy for DFU but also provides novel targets for DFU treatment.

## Data Availability

The datasets presented in this study can be found in online repositories. The names of the repository/repositories and accession number(s) can be found in the article/supplementary material.
